# Hepatoprotective Effect of Fermented Soybean (Nutrient Enriched Soybean Tempeh) against Alcohol-Induced Liver Damage in Mice

**DOI:** 10.1155/2013/274274

**Published:** 2013-08-22

**Authors:** Hamidah Mohd Yusof, Norlaily Mohd Ali, Swee Keong Yeap, Wan Yong Ho, Boon Kee Beh, Soo Peng Koh, Kamariah Long, Suraini Abdul Aziz, Noorjahan Banu Alitheen

**Affiliations:** ^1^Department of Cell and Molecular Biology, Faculty of Biotechnology and Biomolecular Science, Universiti Putra Malaysia, Serdang, Selangor 43400, Malaysia; ^2^Institute of Bioscience, Universiti Putra Malaysia, Serdang, Selangor, Malaysia; ^3^Department of Bioprocess Technology, Faculty of Biotechnology and Biomolecular Science, Universiti Putra Malaysia, Serdang, Selangor 43400, Malaysia; ^4^Biotechnology Research Centre, Malaysian Agricultural Research and Development Institute (MARDI), Serdang, Selangor 43400, Malaysia

## Abstract

Recently, soybean tempeh has received great attention due to many advantages such as higher nutritional value, lower production cost, and shorter fermentation time. In this study, the *in vivo* hepatoprotective and antioxidant effects of nutrient enriched soybean tempeh (NESTE) were determined. NESTE fermentation process which involved anaerobic incubation was previously proclaimed to increase the content of amino acids and antioxidant properties remarkably. The evaluation of histological sections, serum biochemical markers (aspartate aminotransferase (AST), alanine aminotransferase (ALT), and cholesterol and triglycerides (TG)), liver immune response level (nitric oxide (NO)) and liver antioxidant level (superoxide dismutase (SOD), ferric reducing antioxidant power (FRAP), and malondialdehyde (MDA)) was conducted in order to compare the effects of nonfermented soybean extract (SBE) and fermented soybean extract (NESTE) on alcohol-induced liver damage in mice. Results demonstrated that 1000 mg/kg of NESTE can significantly reduce the levels of AST, ALT, cholesterol, TG, MDA, and NO. On the other hand, it also raised the level of SOD and FRAP. Furthermore, the histological examination on 1000 mg/kg NESTE treatment group showed that this extract was capable of recovering the damaged hepatocytes to their normal structures. Thus, it can be concluded that NESTE produced through fermentation process was able to enhance hepatoprotective and antioxidant effects *in vivo*.

## 1. Introduction

 Liver is the largest human's organs after skin and it plays several vital functions including the metabolic, vascular, immunological, secretory, and excretory activities in the body [1]. Liver also provides a major function in the metabolism of fat, protein, and carbohydrate [1]. Numerous substances have been demonstrated to be toxic to the liver cells and alcohol was the most reported cause of liver disease worldwide [2]. 

 Consumption of excessive alcohol can contribute to liver diseases which are mostly initiated by acute inflammation followed by the development of steatosis, alcoholic hepatitis, and eventually cirrhosis and fibrosis [3, 4]. To date, 90% of the heavy alcohol drinkers had developed fatty liver disease (steatosis) and 10% to 20% of these heavy drinkers are suffering from alcoholic hepatitis and cirrhosis [4]. Alcohol-induced liver damage is generally associated with the increasing level of free radicals which cause the development of liver cell peroxidation and would eventually lead to the oxidative stress on the liver cells [5, 6]. Furthermore, the formation of oxidative stress was due to the decreased level of antioxidant defense as the level of free radicals increases [7, 8]. Immune response activity was also reported to be affected in alcohol-induced liver damage where increased level of nitric oxide had been reported [7]. Three different types of histopathological changes were commonly observed and reported on alcohol-induced liver damage, namely, steatosis (fatty liver), steatohepatitis (formerly known as alcoholic hepatitis), and cirrhosis [9]. All of these histopathological changes were linked to the prevalence of oxidative stress which was due to the increase level of reactive oxygen species (ROS) [7, 8]. One of the common histopathological observations for alcohol-induced liver damage was the development of steatosis whereby hepatocytes were occupied with lipid droplets [10]. 

 Several modern medications and treatments are currently available for liver disease patients but, thus far, none of them could fully recover the liver from its pathological conditions [4]. Hence, the search for alternative medicines or supplements with hepatoprotective properties has gained more interest. For example, some studies have shown that soybean has potential bioactive substances that exhibit hepatoprotective properties [4, 11, 12]. Hodgson et al. [13] revealed that soybean might inhibit the oxidation of lipoprotein in serum. Yang et al. [14] have recently reported that soyasaponins-rich extract from soybean may also able to improve on the acute alcohol-induced hepatotoxicity in mice.

 Fermented food, which is one of the alternative curative agents, has been claimed to contain increased level of bioactive constituents [15]. Fermentation processes were found to enhance the nutritional value and efficacy of the soybean [16]. As one of the fermented soybean products, Tempeh has been shown to contain several multifunctional bioactive substances. Furthermore, anaerobic fermented soybean tempeh had been reported to produce substantially higher amount of amino acids and antioxidants than conventional method which involves only aerobic fermentation [17–19]. To date, there was no or little information available on the effect of nutrient enriched soybean tempeh (NESTE) produced through anaerobic fermentation processes against alcohol-induced liver damage in mice model. The present study provides evidence that NESTE gave the best recovery effect against alcohol-induced liver injury in mice models as compared to unfermented soybean extract (SBE).

## 2. Materials and Methods

### 2.1. Materials

Soybeans originated from Canada were purchased from the local markets in Selangor, Malaysia. *Rhizopus 5351 *inoculum was obtained from Malaysian Agricultural Research and Development Institute (MARDI) culture collection centre. Anaerocult A for microbiology and absolute ethanol were purchased from Merck (USA). Standardized milk thistle extract that contained 80% of silybin was obtained from Lipa Pharmaceutical Pty. Ltd., Australia.

### 2.2. Nutrient Enriched Soybean Tempeh Processes

About 1000 g of dehulled soybeans were soaked in cold water at room temperature for 18 h. The soaked soybeans were washed thoroughly and steamed for 40 min. After that, the steamed soybeans were cooled down to room temperature and subsequently mixed with *Rhizopus 5351* inoculum. The inoculated beans were packed into perforated polyethylene plastic bags and incubated aerobically for 30 h at 30°C. Anaerobic incubation was then conducted in order to obtain NESTE. This was carried out by transferring all the packed soybeans into containers containing *Anaerocult A for microbiology* and incubated for 20 h at 30°C. After that, NESTE was dried, ground, and extracted with deionized water. The water extracts of NESTE were then lyophilised using the VirTis BenchTop freeze dryer (SP industries, Inc., USA). Lastly, the lyophilised powder was stored at 4°C in an airtight amber container. The lyophilised extract of NESTE contained 0.338 ± 0.025 g/100 g DW (dried weight) of gamma-aminobutyric acid, 2.176 ± 0.006 g/100 g DW of total free amino acids, 42.64 ± 1.59 *μ*g/g extract of soluble phenolic acids, and 22.56 ± 0.31 mg GAE/g extract of total phenolic acids (manuscript submitted under review). Extract given to the mice was prepared fresh daily by dissolving the lyophilized powder of NESTE into distilled water before administration to the mice.

### 2.3. Animals

Inbred male Balb/c mice aged about 8 weeks old with an average weight of 18–22 g were obtained from the Animal House of Faculty of Veterinary, Universiti Putra Malaysia. The mice were placed in plastic cages at room temperature (22 ± 1°C) with 60% relative humidity and 12 h of dark/light cycle. The mice were fed with standard pellet diet and supplied with distilled water *ad libitum*. These animals were acclimatized for 7 days prior to the study. The liver protection study was conducted under the approval of Animal Care and Use Committee, Universiti Putra Malaysia (UPM) (UPM/FPV/PS/3.2.1.551/AUP-R2).

### 2.4. Experimental Design

The male Balb/c mice were randomly divided into seven groups with eight mice each. Ethanol and extract treatment of mice was performed according to previous study [20]. For liver damage induction, the mice from all groups except for the normal control (group I) were treated with 50% (v/v) ethanol at a dose of 2 g/kg via gavage for 7 d. After that, all mice were subjected to the treatments preorally once daily for 14 d via gavage as follows:

*Group I:* normal control group without ethanol induction receiving only phosphate buffer saline solution,

*Group II:* ethanol control group receiving phosphate buffer saline solution,

*Group III*: positive control group receiving 50 mg/kg of Silybin extract,

*Group IV*: treatment group receiving 200 mg/kg of soybean extract (SBE), 

*Group V:* treatment group receiving 1000 mg/kg of SBE,

*Group VI:* treatment group receiving 200 mg/kg of NESTE,

*Group VII:* treatment group receiving 1000 mg/kg of NESTE.

At the end of the experimental period, all the mice were sacrificed after isoflurane anaesthesia. Blood was collected via cardiac puncture from each mouse and subjected to quantification of aspartate aminotransferase (AST), alanine aminotransferase (ALT), cholesterol, and triglycerides (TG) concentrations in the serum. Then, the liver was excised from each mouse and weighed. The organs were meshed through 0.2 *μ*m cell strainer (SPL Life Sciences, China) using syringe rubber plunger in cold phosphate-buffered saline solution. The liver homogenate was then used to determine the level of superoxide dismutase (SOD), malondialdehyde (MDA), ferric reducing antioxidant power (FRAP), and nitric oxide (NO). Mice liver was placed in 10% neutral buffered formaldehyde solution for histopathological examination. 

### 2.5. Analyses of Liver Homogenate (MDA, SOD, NO, and FRAP Assay)

Malondialdehyde (MDA) formation in the liver, which is a thiobarbituric acid-reactive substance, was measured by lipid peroxidation estimation according to Suhail et al. [21] with slight modification. The estimation of MDA level in liver homogenate was performed by mixing 100 *μ*L of liver homogenate sample with 400 *μ*L of phosphate-buffered saline, 12.5 *μ*L of butylated hydroxytoluene (8.8 mg/mL absolute ethanol), and 250 *μ*L of 30% w/v trichloroacetic acid. The mixed solution was then vortexed and put on ice for approximately 2 h. After that, the solution was centrifuged at 2000 g and 25°C for 15 min. 500 *μ*L of supernatant was then mixed with 37.5 *μ*L of 0.1 M EDTA and 125 *μ*L of 1% v/v thiobarbituric acid in 0.1 M NaOH. The mixed solution was boiled for 15 min and cooled down to room temperature. The aliquot of 100 *μ*L of solution was finally read *via* ELISA plate reader at 532 nm and 600 nm. 

The activity of superoxide dismutase (SOD) was measured according to Ilouno et al. [22]. The assay was performed in 96 well plate by loading 10 *μ*L of liver homogenate sample into each well with variable concentrations. The liver sample homogenate was then mixed with 290 *μ*L of SOD reagent. The SOD reagent consisted of 0.015 M sodium cyanide, 1.5 mM nitroblue tetrazolium, 0.012 M riboflavin, and 0.067 M phosphate buffer (4: 2: 1: 51). The reaction solution was then read at 560 nm after 5 min of incubation. 

The total antioxidant capacity in the liver was determined by ferric reducing antioxidant power (FRAP) assay according to Benzie and Strain [23] with some modifications. Ferum sulphate (Fe^II^) calibration curve was constructed with concentrations range from 100 to 1000 *μ*mol/L. In each 96 well plate, 20 *μ*L of liver homogenate sample was loaded and added with 60 *μ*L of distilled water. The sample solution was then mixed with the FRAP working solution which consists of 15 mL acetate buffer, 1.5 mL TPTZ solution, and 1.5 mL FeCl_3_·6H_2_O. The reaction mixture was measured using ELISA plate reader at 593 nm for every 2 min up to 10 min. 

Nitric oxide (NO) inhibition assay was conducted using Griess reagent kit for nitrite determination (Molecular Probes, Inc., USA).

### 2.6. Clinical Chemistry Analyses

All serum biochemical activity such as alanine aminotransferase (ALT), aspartate aminotransferase (ASP), cholesterol, and triglyceride (TG) was measured by using standard assay kits (Roche Diagnostics GmbH, USA) and analyzed using the 902 automatic analyzer (Hitachi-Boehringer Mannheim).

### 2.7. Histopathological Examination

The liver tissues were preserved in 10% buffered formaldehyde solution and then stained with hematoxylin and eosin (H and E) according to Luna [24]. The method was initially done by embedding the organs with paraffin wax prior to cutting process via microtome. The sliced organs which had been deparaffinised were then stained with hematoxyline and eosin. The stained organs were ultimately observed under a *Nikon Eclipse 90i* microscope (New York, USA) by Bright field optics with 100 times magnification.

### 2.8. Statistical Analysis

The significance value of all the data was analyzed using one-way analysis of variants (ANOVA) from SPSS 15 software. Duncan's multiple range test was used and *P* value less than 0.05 was considered significant.

## 3. Results 

### 3.1. Concentrations of ALT, AST, Cholesterol, and Triglyceride in Mice Serum

Table 1 illustrates the level of biochemistry profile of different experimental groups. The levels of ALT and AST in ethanol control group were increased significantly as compared to other experimental groups. The high level of AST and ALT was due to the liver cell injuries induced by alcohols. The levels of AST and ALT in NESTE (200 mg/kg and 1000 mg/kg body weight) and SBE (200 mg/kg and 1000 mg/kg body weight) were significantly lower in values than the ethanol control group. Among all, NESTE at 1000 mg/kg body weight was the most effective concentration that restrained the level of AST and ALT.

The cholesterol and triglyceride (TG) level of untreated ethanol control group was significantly higher than the other experimental groups. In contrast, the levels of cholesterol and TG of the NESTE groups were significantly lower than the ethanol control group. Added to this, 1000 mg/kg of SBE and both NESTE concentrations were able to reduce the level of cholesterol and TG to near the normal value.

### 3.2. MDA, NO, SOD, and FRAP Level in Liver Homogenate

The measurement of MDA, NO, SOD, and FRAP level on liver homogenate was conducted based on the amount of liver total protein and the results were demonstrated in Table 2.

#### 3.2.1. MDA Content on Liver Homogenate

The amount of MDA in ethanol control group was significantly increased as compared to other experimental groups. The increased amount of MDA in ethanol control group had shown the occurrence of substantial lipid peroxidation of liver cells which was due to the toxic effect of alcohol. Furthermore, all of the extract treatment groups were significantly different (*P* < 0.05) as compared to ethanol control group and the MDA content was normalized as compared to normal control and ethanol control groups. Both extracts of 1000 mg/kg SBE and NESTE had contributed to the lower amount of MDA as compared to normal control. Among all the treatment groups, 1000 mg/kg NESTE shows the lowest production of MDA (1.34 ± 0.23 nmol/g protein).

#### 3.2.2. NO Level on Liver Homogenate

Nitric oxide (NO) level of the ethanol control group was significantly increased as compared to other experimental groups. This result confirms the sign of substantial liver cells injuries which was predicted to occur in the ethanol control. The extract treatment groups of SBE and NESTE at both concentrations also showed significant difference (*P* < 0.05) as compared to ethanol control groups where their NO level was lower and comparable to the normal and positive control. Nevertheless, high concentration of NESTE at 1000 mg/kg showed the lowest level of NO which was 8.45 ± 0.27 *μ*M/mg protein.

#### 3.2.3. SOD Level on Liver Homogenate

As compared to ethanol control group, all of the treatment groups showed significant difference (*P* < 0.05) and produced higher level of SOD. All of the extract treatment groups including the positive control group showed significantly higher SOD level as compared to the normal control. The highest SOD level was illustrated from both SBE and NESTE of 1000 mg/kg which were 20.08 ± 1.58 U/mg protein and 20.53 ± 0.24 U/mg protein, respectively.

#### 3.2.4. FRAP Activity on Liver Homogenate

All of the treatment groups including normal and positive control groups were significantly different with ethanol control group. Based on the results, mice treated with 1000 mg/kg of NESTE showed a significantly higher level of FRAP as compared to ethanol control group which was 12.78 ± 0.10 mM  Fe^II^/mg protein while mice treated with 200 mg/kg of NESTE were shown to normalize the FRAP value. In contrary, both SBE-treated groups gave a lower level of FRAP value as compared to normal control group. These results indicated that 1000 mg/kg of NESTE could give a higher level of FRAP value even though this value was lower than the positive control group. 

### 3.3. Histopathological Examination

**Figure 1:**
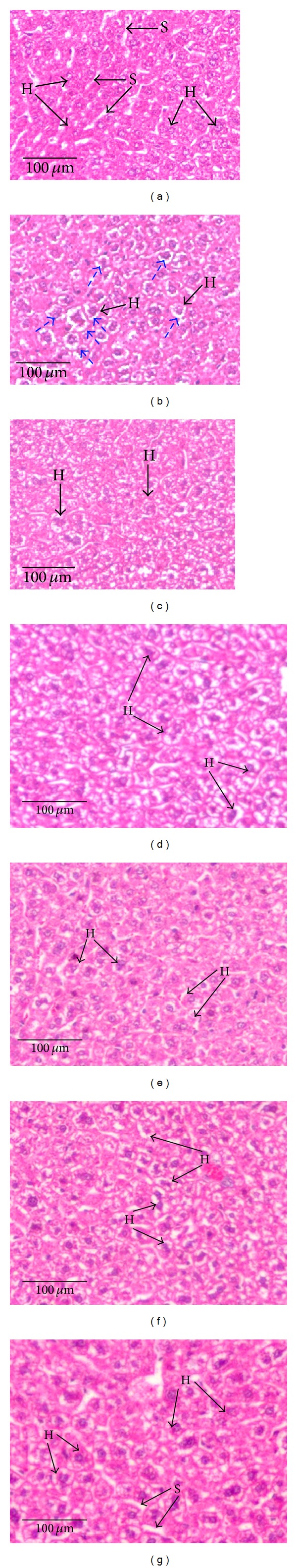
Histopathological microsection of liver of different experimental groups. The histopathological microsection of liver was stained with hematoxylin and eosin and captured via the light microscope with the magnification of 10 × 10. (a) Normal control with normal structure of liver cells, (b) ethanol control with a several-hepatocyte condition which has developed a microvesicular steatosis as indicated by the dashed line arrows, (c) positive control with a minimal condition of microvesicular steatosis, (d) 50% ETOH + 200 mg/kg SBE-treated group with the obvious development of microvesicular steatosis, (e) 50% ETOH + 1000 mg/kg SBE-treated group with recovery effect from microvesicular steatosis, (f) 50% ETOH + 200 mg/kg NESTE-treated group with some obvious development of microvesicular steatosis, and (g) 50% ETOH + 1000 mg/kg NESTE-treated group with recovery effect from microvesicular steatosis. H = hepatocyte, S = sinusoidal space, ⇢  hepatocyte developed with microvesicular steatosis.

Histopathological observation was based on the liver section from different experimental groups which had been stained with H and E staining and observed *via* a light microscope (Figure 1). Normal hepatocytes were observed in normal control group (1A) while, for the ethanol control (1B), the hepatocytes had developed fatty changes which were also known as microvesicular steatosis where the hepatocytes developed an open space around the nuclei. Positive control (silybin extract 50 mg/kg) (1C) showed the hepatocytes condition having a recovery with minimal microvesicular steatosis. 1000 mg/kg of SBE and both NESTE concentrations showed a recovery effect of hepatocytes with minimal to no microvesicular steatosis.

## 4. Discussion

Alcoholic liver disease is associated with several reactions of free radicals such as reactive oxygen species (ROS) which then contribute to the augmentation of MDA and NO. Furthermore, the level of SOD and FRAP falls tremendously along with the rise of ROS. This condition as well could lead to the rise of serum biochemical markers such as AST, ALT, triglyceride, and cholesterol. Liver steatosis was normally found in alcoholic liver histology and it was marked as a primary indicator for alcoholic liver disease. NESTE was claimed to be enriched with amino acids and antioxidants might serve as a curable agent towards alcoholic liver disease where it could revert and lower the free radicals level. 

In the present study, we had evaluated the hepatoprotective effect of NESTE and SBE against the ethanol-induced liver damage in mice. In this investigation, 1000 mg/kg of NESTE treatment had the ability to reduce the serum alanine transaminase (ALT) and aspartate transaminase (AST) level. Similar trend was observed for the serum cholesterol and triglycerides level where 1000 mg/kg of NESTE was able to reduce the level of these parameters in ethanol-induced mice. The rise of serum enzyme ALT and AST level had indicated that hepatocyte cells were injured where the leakage of cell membrane had contributed to the accumulation of these enzymes into the plasma [2, 25]. Therefore, prolonged treatment of 1000 mg/kg of NESTE administration could help to normalize the ALT and AST enzyme levels. In addition, significant reduction of triglycerides and cholesterol was in agreement with the previous studies where soybeans and soybean-based products have been revealed to have hypocholesterolaemic effect [4, 16]. 

Malondialdehyde (MDA) is known as a secondary metabolite that is produced from the lipid peroxidation of cell membrane and the amount of MDA is used as an indicator for lipid peroxidation of cell membrane which could cause cell damage [21]. In contrast, nitric oxide (NO) plays the role as an inflammatory indicator where it would accumulate as the hepatocytes injuries [26]. The levels of NO and MDA had reduced in both alcohol-induced liver damage treated with 1000 mg/kg of NESTE and SBE which suggested that both of these extracts had shown the protective and curative activities against liver injury *via* alcoholic intoxication. However, treatment with 1000 mg/kg of NESTE possessed the most reduction level of MDA and NO in alcohol-induced liver damage in mice. On the other hand, these results were in agreement with the previous investigations where soybean had been reported to reduce the MDA level on CCl_4_-induced liver in rats [27]. Other previous study also showed that the fermented soybean milk which is one of soybean-based product could alleviate and protect the injured cells from the rise of NO level [28]. The findings from these studies were comparable to our investigation where alcohol-induced liver damage in mice treated with 1000 mg/kg of NESTE and SBE could reduce the level of MDA and NO.

The level of superoxide anions which is one of the reactive oxygen species (ROS) would arise as the cell injured or inflamed. Superoxide dismutase (SOD) enzyme was found naturally in most of the organ cells and basically protects the membrane cells from damage by harmful superoxide-free radicals [22, 29]. The decreased level of SOD indicates that the high risks of cell injuries and association with supplement or highly nutritious foods tend to increase or normalize the level of SOD. The level of SOD in the liver of alcohol-induced mice treated with both 1000 mg/kg of NESTE and SBE was observed to be increased. However, treatment of 1000 mg/kg of NESTE in alcohol-induced liver damage in mice showed the highest level of SOD as compared to 1000 mg/kg of SBE. In addition, conventional tempeh had been reported previously to have SOD enzyme [30], and this signified that NESTE could provide the elevated SOD enzyme to the injured liver cells which in turn could recover the liver cells to normal and eventually be able to produce the significant amount of SOD enzyme by itself as a protective action from the damage caused by toxic substance such as alcohol. 

The bioavailability and antioxidant level in the alcohol-induced liver damage in mice treated with 1000 mg/kg of NESTE was increased demonstrated by the significant increase of FRAP value. This suggested that the extract may have a significant amount of antioxidant in order to recover the liver cell damage caused by alcohol intoxication. This result could also be correlated to the previous investigation where soybean tempeh was known to have substantial amount of antioxidant properties [4, 11, 12, 31, 32]. Our result was also comparable to the positive control (50 mg/kg silybin extract) since the FRAP value from positive control treatment was slightly higher than the treatment of 1000 mg/kg of NESTE.

Histological sections (Figure 1) present the comparative appearances of hepatocytes of the treated and untreated mice from alcohol intoxication.  Figure 1(a) showed a normal control group with normal hepatocytes structure. Liver histology of ethanol control (Figure 1(b)) showed a clear damage with association of fatty liver degeneration which was also known as steatosis. The mechanism of steatosis was initially associated with the damage of hepatocytes structure such as mitochondria and reticulum endoplasmic. This damage inhibits the oxidation of fatty acid which would eventually accumulate in the hepatocytes [10]. The liver condition, as in Figure 1(b), was observed to be in a stage of microvesicular steatosis where the hepatocytes had loaded with excessive lipid droplets without dislocation of the nucleus to the periphery [10]. The severity of microvesicular steatosis could lead to the development of macrovesicular steatosis which has an opposite characteristic of microvesicular steatosis where the nucleus would dislocate and develop a single large vacuole of fat [10]. A minimum recovery from microvesicular steatosis was observed when treated with the positive control silybin extract (50 mg/kg) (Figure 1(c)), which is well-known to have substantial antioxidant and hepatoprotective effects [33]. The dissimilarity condition of hepatocytes between Figures 1(d) (200 mg/kg of SBE) and 1(e) (1000 mg/kg of SBE) was observed where hepatocytes condition in Figure 1(e) showed a recovery from microvesicular steatosis. This condition was also shown in Figures 1(f) (200 mg/kg of NESTE) and 1(g) (1000 mg/kg of NESTE) where the recovery from microvesicular steatosis was observed in Figure 1(g). As a result, the 1000 mg/kg of SBE and NESTE may have provided a recovery effect to hepatocytes from having a microvesicular steatosis. In addition, these histopathological findings could be linked to all of other results of our study where 1000 mg/kg of NESTE gave the best recovery effect against alcohol-induced liver cell injury among all other treatment groups.

## 5. Conclusion

Overall, the administration of 1000 mg/kg of NESTE demonstrated an effective amelioration against alcohol-induced liver. These results also showed that 1000 mg/kg of NESTE was not only comparable but was more effective than the positive control (silybin 50 mg/kg) in reverting the serum biochemical markers (AST and ALT), lipid profile (cholesterol and triglycerides), liver homogenate test (SOD, MDA, NO, and FRAP), and histological conditions back to normal. This effect was in agreement with the hypothesis that soybeans could ameliorate the liver cell damage and NESTE is one type of soybean-based products. Further investigation such as combination of *in vitro* works would offer more understanding about the mechanism of amelioration of alcohol-induced liver damage *via* administration of NESTE.

## Figures and Tables

**Table 1:** Serum biochemical parameter of liver protective effect of different experimental group.

Treatment	ALT (U/L)^1^	AST (U/L)^1^	Cholesterol (mmol/L)^1^	TG (mmol/L)^1^
Normal control (PBS)	14.09 ± 1.53^a^	98.16 ± 1.99^a^	3.14 ± 0.39^a^	1.48 ± 0.23^a^
Ethanol control (50% ETOH + PBS)	48.11 ± 1.78	367.30 ± 1.10	4.83 ± 0.3	3.87 ± 0.3
Positive control (50% ETOH + silybin (50 mg/kg))	26.72 ± 1.20^a^	171.70 ± 3.79^a^	4.11 ± 0.8^a^	2.53 ± 0.4^a^
50% ETOH + SBE (200 mg/kg)	35.14 ± 4.50^a^	183.28 ± 7.28^a^	3.26 ± 0.11^a^	2.16 ± 0.16^a^
50% ETOH + SBE (1000 mg/kg)	25.85 ± 6.49	174.49 ± 1.15	2.99 ± 0.29^a^	2.03 ± 0.01^a^
50% ETOH + NESTE (200 mg/kg)	23.28 ± 4.50^a^	168.39 ± 3.42^a^	3.22 ± 0.26^a^	2.88 ± 0.26^a^
50% ETOH + NESTE (1000 mg/kg)	18.50 ± 3.00^a^	157.62 ± 3.53^a^	3.08 ± 0.44^a^	1.94 ± 1.85^a^

^1^Values are expressed as mean ± S.E.M (*n* = 8), ^a^values are significantly different from ethanol control (*P* < 0.05), ALT: alanine transaminase, AST: aspartate transaminase, TG: triglyceride.

**Table 2:** Liver protective effect on liver homogenate parameters of different experimental groups.

Treatment	MDA (nmol/g protein)^1^	NO (*µ*M/mg protein)^1^	SOD (U/mg protein)^1^	FRAP (mM FeII/mg protein)^1^
Normal control (PBS)	3.20 ± 0.22^a^	10.13 ± 0.18^a^	17.17 ± 0.74^a^	9.42 ± 1.0^ a^
Ethanol control (50% ETOH + PBS)	7.25 ± 0.08	15.09 ± 0.86	12.46 ± 2.51	5.28 ± 0.01
Positive control (50% ETOH + silybin (50 mg/kg))	4.95 ± 0.08^a^	10.20 ± 3.08^a^	18.54 ± 0.37^a^	15.02 ± 0.07^a^
50% ETOH + SBE (200 mg/kg)	3.02 ± 0.25^a^	9.55 ± 0.35^a^	19.38 ± 0.10^a^	6.59 ± 0.03
50% ETOH + SBE (1000 mg/kg)	2.93 ± 0.34^a^	9.54 ± 0.04^a^	20.08 ± 1.58^a^	6.24 ± 0.04
50% ETOH + NESTE (200 mg/kg)	2.22 ± 0.17^a^	9.55 ± 0.58^a^	19.44 ± 0.67^a^	9.42 ± 0.02^a^
50% ETOH + NESTE (1000 mg/kg)	1.34 ± 0.23^a^	8.45 ± 0.27^a^	20.53 ± 0.24^a^	12.78 ± 0.10^a^

^1^Values are expressed as mean ± S.E.M (*n* = 8), ^a^values are significantly different from ethanol control (*P* < 0.05), MDA: malondialdehyde, NO: nitric oxide, SOD: superoxide dismutase, FRAP: ferric reducing antioxidant power.
